# Phlorizin Alleviates Depression-like Behaviors via Gut Microbiota Reprogramming-Induced Methionine to Inhibit Neuroinflammation in Mice Hippocampus

**DOI:** 10.3390/ph18091395

**Published:** 2025-09-17

**Authors:** Lingling Li, Jianxin Chen, Xinyu Zhang, Xuya Zhang, Yan Fu, Hong Jiang, Tianxing Yin, Yali Zhang, Xue Li, Mengyuan Hu, Yi Lu

**Affiliations:** School of Traditional Chinese Medicine, Beijing University of Chinese Medicine, Beijing 102488, China; 20240941084@bucm.edu.cn (L.L.); cjx@bucm.edu.cn (J.C.); 18811385506@163.com (X.Z.); 20220931137@bucm.edu.cn (X.Z.); 20230931133@bucm.edu.cn (Y.F.); 18282643481@163.com (H.J.); ytx18515831039@163.com (T.Y.); zhangyalichn@163.com (Y.Z.); crzay9@126.com (X.L.); hmy19942021@163.com (M.H.)

**Keywords:** depression, phlorizin, gut microbiota, methionine, microbiota–gut–brain axis

## Abstract

**Background**: Depression is associated to gut microbiota imbalance. Our research examined the antidepressant potential of phlorizin (PHZ), a natural anti-inflammatory compound that influences gut microbiota, and explored its underlying mechanisms. **Methods**: A corticosterone (CORT)-induced depression mouse model was used for evaluating the ameliorative influences of PHZ on depressive phenotypes and central neuroinflammation through behavioral tests and biochemical assays. 16S rRNA sequencing and metabolomics were used to evaluate gut microbiota composition and metabolite levels in serum and hippocampal tissue, respectively. Spearman correlation and broad-spectrum antibiotic cocktail (ABx) treatment experiments verified the effect of gut microbes in the PHZ-mediated modulation of key metabolites. A lipopolysaccharide (LPS)-induced BV2 microglial inflammation model was established to evaluate the role of metabolites in PHZ’s antineuroinflammatory effects. **Results**: PHZ significantly alleviated depressive-like behaviors in CORT mice and suppressed hippocampal neuroinflammation by modulating microglial M1/M2 polarization. Furthermore, PHZ altered gut microbiota composition, influenced serum methionine (Met) metabolism, and significantly increased hippocampal L-methionine (L-Met) and S-adenosylmethionine (SAMe) levels. Cellular experiments confirmed that L-Met plays a critical role in PHZ-mediated antineuroinflammatory effects. Significant correlations were observed between Parabacteroides, Parasutterella, and Alistipes and serum Met levels. ABx treatment suppressed the increase in hippocampal L-Met levels, suggesting that PHZ regulates methionine metabolism via the microbiota. These findings indicate that PHZ alleviates depressive states in CORT mice by modulating the microbiota–gut–brain axis. **Conclusions**: PHZ modulates the gut microbiota (namely *Parabacteroides*, *Parasutterella*, and *Alistipes*) and increase L-Met and SAMe levels, thereby suppressing neuroinflammation and improving depressive phenotypes in mice.

## 1. Introduction

Depression is a complex, recurrent psychiatric disorder characterized by low mood, loss of interest, fatigue, and impaired cognitive function [1]. Recently, the incidence of depression has been increasing among younger populations [2]. The World Health Organization projects depression as a leading global burden by 2030 [3]. However, current clinical antidepressants targeting known etiological factors, like tricyclic antidepressants and selective serotonin reuptake inhibitors (SSRIs), are restricted through delayed onset, efficacy, and significant side effects [4]. Consequently, there is an urgent demand for developing safer, more effective antidepressants based on novel pathophysiological mechanisms to improve clinical outcomes and remission rates.

The microbiota–gut–brain (MGB) axis has emerged as a pivotal pathway in central nervous system disorders [5]. Gut microbiota serve as key mediators between environmental factors and host health, and have been closely linked to depression [6]. Transfer of gut microbiota from patients with depression to germ-free mice induces depressive-like behaviors and alters brain function, metabolites, and proteins via the MGB axis [7]. For instance, depressed mice exhibit dysregulated carbohydrate and amino acid metabolism owing to microbial gene dysfunction [8]. Notably, microbiota-mediated metabolic remodeling can exacerbate the pathological impact on the central nervous system via neuroinflammatory mechanisms [9]. Neuroinflammation, typically accompanied by heightened microglial activation in depression-related brain regions [10], is considered a key pathological hallmark of depression [11]. A novel perspective posits that the MGB axis might be crucial to comprehend depression and develop novel antidepressant therapies [12].

Phlorizin (PHZ; C_21_H_24_O_10_), a small phenolic compound isolated from plants, exhibits diverse pharmacological properties, like antioxidant, antidiabetic, antitumor, antiobesity, antibacterial, and antiviral activities [13,14]. Recently, evidence highlighted an association between restoring gut microbial community balance and treating neurodegenerative and metabolic diseases using PHZ. Chen et al. reported that 20 mg/kg PHZ improved gut microbiota composition and modulated NF-κB signaling in D-galactose-induced aging mice [15], while Zhang et al. found that PHZ reshaped gut microbiota and suppressed hippocampal neuroinflammation in high-fat, high-fructose diet mice [16]. Thus, we propose that PHZ may exert central pharmacological effects via a “periphery-to-center” mechanism, in which gut microbiota regulation potentially serves as a key mechanism for its “remote” antidepressant effects.

Gut microbiota influence brain function partly through small-molecule metabolites, a process commonly studied by metabolomics [17,18,19,20]. Integrating brain and peripheral blood metabolomics with gut microbiota alterations can provide deeper perceptions of the efficacy of the MGB axis in the antidepressant efficacy of PHZ. In this study, we used a corticosterone (CORT)-induced depression model to preliminarily investigate the efficacy of PHZ in ameliorating depressive phenotypes and neuropathology. We further examined its effects on microglial M1/M2 polarization and the expression of inflammatory cytokines. In addition to examining gut microbiota composition, we analyzed serum and hippocampal metabolism. To verify the role of gut microbiota in regulating methionine (L-Met) metabolism, we employed broad-spectrum antibiotic (ABx) treatment. Finally, a lipopolysaccharide (LPS)-induced BV2 cell model was used to confirm the contribution of L-Met to PHZ’s antineuroinflammatory effects.

## 2. Results

### 2.1. Oral PHZ Improves CORT Mice’s Weight and Behavior

**Figure 1:**
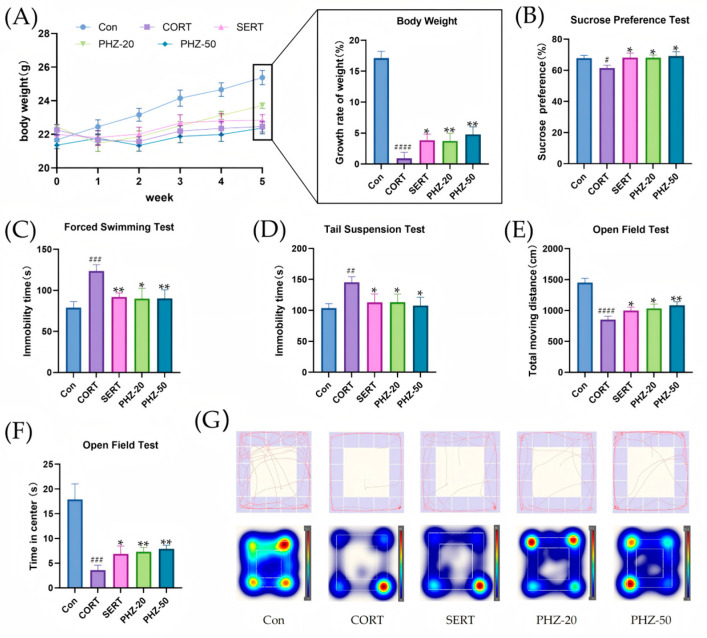
Corticosterone (CORT)-induced depression-like behaviors in mice. (**A**) Body weight change and growth rate; (**B**) Sucrose preference test (SPT); (**C**) Immobility time in the forced swim test (FST); (**D**) Immobility time in the tail suspension test (TST); (**E**,**F**) Open field test (OFT) data analysis; (**G**) OFT track diagram and heatmap. Data are expressed as mean ± SEM, *n* = 9–10. # *p* < 0.05, ## *p* < 0.01, ### *p* < 0.001, #### *p* < 0.0001 compared with Control group; * *p* < 0.05, ** *p* < 0.01 compared with CORT group.

Mouse body weights were determined per week over a 5-week period to appraise the influence of PHZ (Figure 1A). As to the CORT-treated mice, the body weight gradually decreased relative to that of controls, indicating that CORT may affect body weight in growing mice. Throughout the medication administration period, the mice in all treatment groups experienced a significantly greater rate of weight gain than the model group. It is noteworthy that our findings indicate that PHZ exerts a similar effect to sertraline on counteracting CORT-induced weight variations. Depressive-like behaviourbehaviors were assessed using the sucrose preference test (SPT), immobility time in the forced swim test (FST), immobility time in the tail suspension test (TST), and open field test (OFT). CORT-induced depression caused anhedonia and despair, as reflected by a decrease in SPT and a rise in immobility time relative to controls (Figure 1B–D). An obvious decrease in immobility duration in both the TST and FST after the administration of PHZ indicates the PHZ’s therapeutic potential. In the OFT (Figure 1E–G), mice with CORT-induced depression exhibited reduced locomotor activity, and central area retention time than controls. In contrast, PHZ increased the total distance moved and time spent in the centrecenter after 5 weeks of administration compared with the CORT group, suggesting that PHZ alleviated CORT-induced depression.

### 2.2. PHZ Decreased the Neuroinflammation in CORT Mice

**Figure 2:**
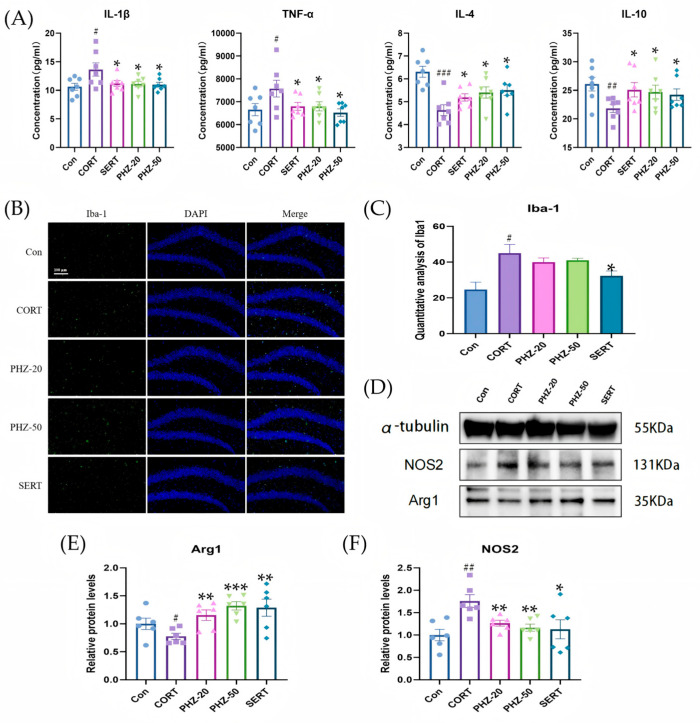
Phlorizin (PHZ) can induce the transformation of microglia-related proteins in the hippocampus of depressed mice and relieve the inflammatory response. (**A**) The levels of IL-1β, TNF-α, IL-4, and IL-10 were measured by enzyme-linked immunosorbent assay (ELISA, *n* = 7); (**B**) Representative images of Iba-1-positive cells (green) in the hippocampus (Scale bar = 100 μm); (**C**) Quantification of Iba-1 immunostaining in hippocampal sections from different groups (*n* = 3); (**D**) Western blot analysis of NOS2 and Arg1 expression, with α-tubulin as loading control; (**E**,**F**) Densitometric analysis of NOS2 and Arg1 protein levels. Data are expressed as mean ± SEM, *n* = 6. # *p* < 0.05, ## *p* < 0.01, ### *p* < 0.001 compared with Control group; * *p* < 0.05, ** *p* < 0.01, *** *p* < 0.001 compared with CORT group.

To appraise the influence of PHZ on neuroinflammation among CORT-treated mice, we first measured the levels of anti-inflammatory cytokines (IL-4 and IL-10) and pro-inflammatory cytokines (IL-1β and TNF-α) within the hippocampus via ELISA. CORT treatment remarkably raised the expressions of pro-inflammatory cytokines and decreased the levels of anti-inflammatory cytokines, relative to controls. By contrast, PHZ treatment decreased the expressions of IL-1β and TNF-α, while increasing IL-10 and IL-4 levels in a dose-dependent manner (Figure 2A). The above finding indicates that PHZ effectively modulates the inflammatory response in the hippocampus of mice suffering from CORT-induced depression. Hence, the anti-inflammatory effects of PHZ prompted us to further investigate its role in microglial activation and polarization. To investigate PHZ’s potential role in microglial activation, immunofluorescence staining for the microglial marker Iba1 was performed (Figure 2B). CORT treatment brought about the obviously heightened quantity of activated Iba-1^+^ microglia within the hippocampus. However, PHZ treatment maintained Iba-1^+^ microglia numbers comparable to controls, indicating that PHZ attenuated CORT-induced microglial activation (Figure 2C). We further investigated the effects of PHZ on microglial polarization. Western blot analysis was used to measure NOS2 (M1 marker) and Arg1 (M2 marker) levels in the hippocampus (Figure 2D–F). In the CORT-treated group, a marked increase in NOS2 levels and reduction in Arg1 levels were observed, indicating a shift towards M1 polarization. In contrast, PHZ treatment significantly decreased NOS2 levels and increased Arg1 levels, suggesting that PHZ promoted M2 polarization and reduced M1 activation in the hippocampus. These results demonstrate that PHZ effectively suppresses abnormal microglial activation, modulates the polarization, and reduces neuroinflammation.

### 2.3. PHZ Restored the Gut Microbiota of CORT Mice

**Figure 3:**
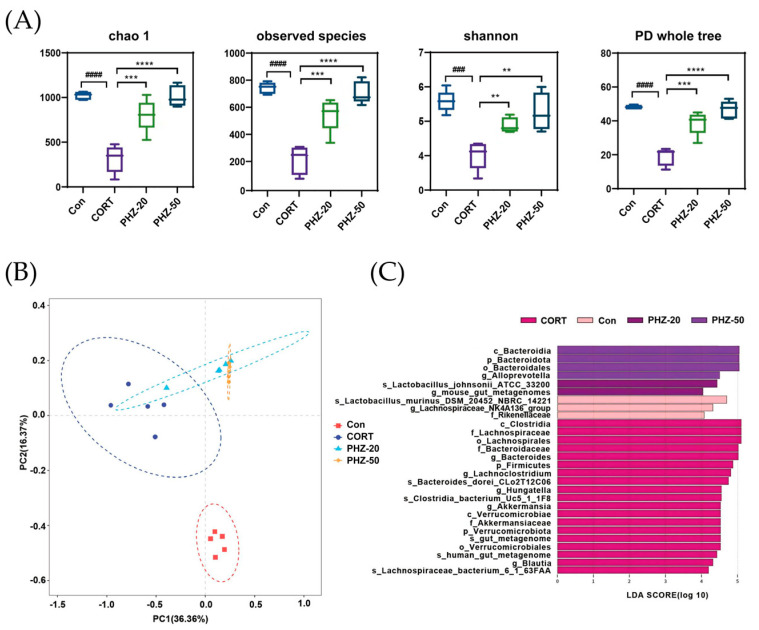
Phlorizin (PHZ) altered the gut microbiota composition of corticosterone (CORT) mice. (**A**) Alpha diversity was assessed using Chao 1 index, observed species, Shannon index, and phylogenetic diversity (PD) whole tree; (**B**) Beta diversity was evaluated using Bray–Curtis UniFrac-based principal coordinate analysis (PCoA) across groups; (**C**) Potential gut microbiota biomarkers were identified by linear discriminant analysis effect size (LEfSe). The histogram shows the linear discriminant analysis (LDA) scores for differentially abundant taxa (threshold = 2.0). Taxonomic ranks are indicated as follows: c, class; o, order; f, family; g, genus. ### *p* < 0.001, #### *p* < 0.0001 compared with Control group; ** *p* < 0.01, *** *p* < 0.001, **** *p* < 0.0001 compared with CORT group.

**Figure 4:**
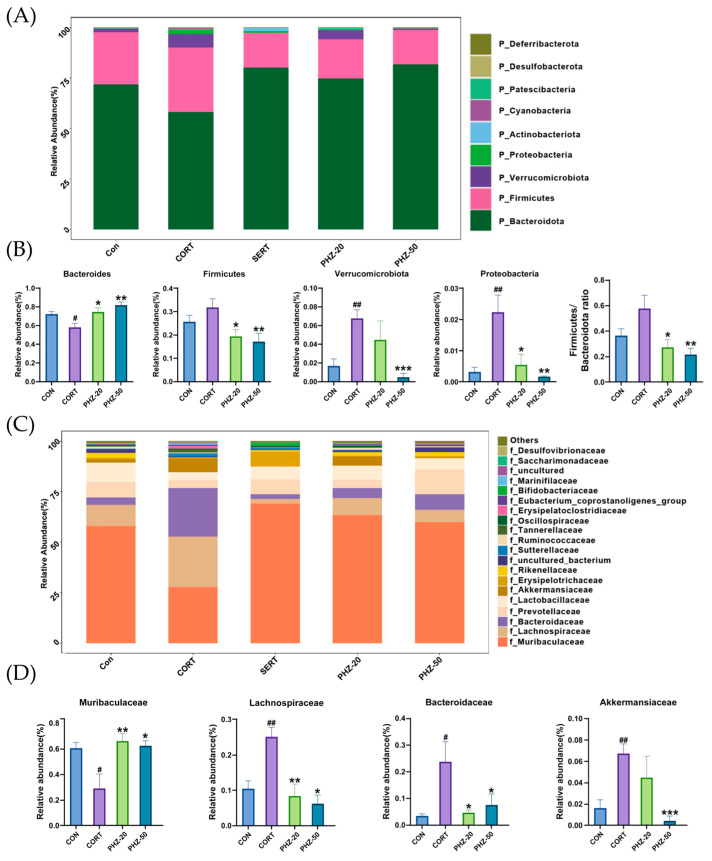
Compositional analysis of gut microbiota among groups at the phylum and family levels. (**A**) The relative abundances of gut microbiota at the phylum level; (**B**) The significant changes of the representative phylum after phlorizin treatment; (**C**) The relative abundances of gut microbiota at the family level; (**D**) The significant changes of the representative family after phlorizin treatment. Data are expressed as mean ± SEM, *n* = 5. # *p* < 0.05, ## *p* < 0.01 compared with Control group; * *p* < 0.05, ** *p* < 0.01, *** *p* < 0.001 compared with CORT group.

**Figure 5:**
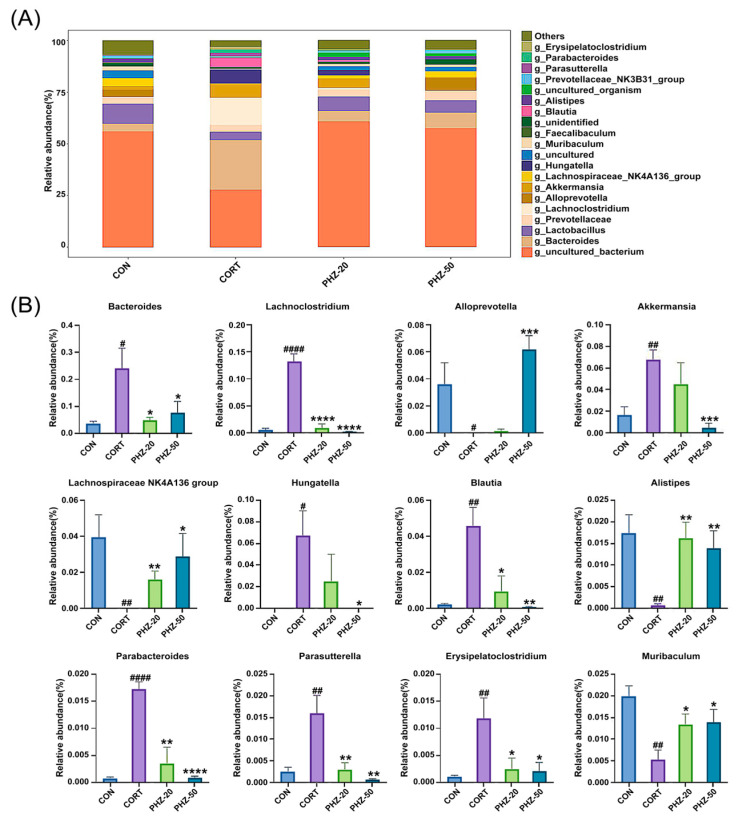
Compositional analysis of gut microbiota among groups at the genus levels. (**A**) Relative abundances of gut microbiota at the genus level; (**B**) Significant changes of the representative genus after phlorizin treatment. Data are expressed as mean ± SEM, *n* = 5. # *p* < 0.05, ## *p* < 0.01, #### *p* < 0.0001 compared with Control group; * *p* < 0.05, ** *p* < 0.01, *** *p* < 0.001, **** *p* < 0.0001 compared with CORT group.

**Figure 6:**
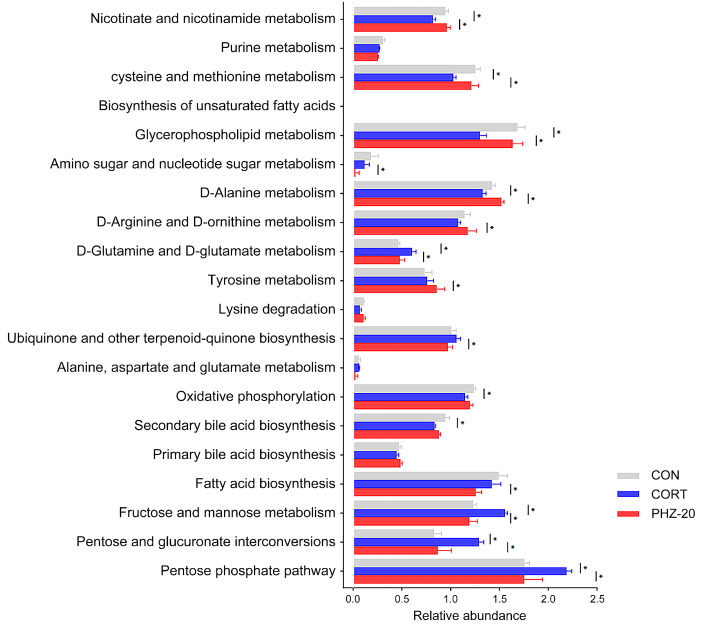
STAMP analysis of predicted microbial functions based on Phylogenetic Investigation of Communities by Reconstruction of Unobserved States 2 (PICRUSt2) among Control, corticosterone (CORT), and phlorizin 20 mg/kg (PHZ-20) groups. * *p* < 0.05.

To explore whether the efficacy of PHZ on depression is related to the gut microflora, the microbial composition of fecal samples from each experimental group was analyzed by 16S rRNA sequencing. As indicated by the α diversity indices (Figure 3A), CORT treatment resulted in significantly lower Chao1, Shannon, and Simpson PD indices, reflecting reduced microbial richness and diversity, which corresponds to former reports on gut dysbiosis in depression models [21,22]. PHZ reversed these indicators, increasing microbial richness and diversity. Beta diversity further showed remarkable disparities in microbial communities between CORT and control mice (Figure 3B), suggesting a shift in the microbiota composition due to CORT treatment. PHZ-50 intervention affected the largest number of bacterial genera and modulated the host gut microbiota more efficiently (Figure 3C), restoring microbial balance in the CORT mice. The relative abundances (RAs) of the gut microflora were enumerated at the phylum level (Figure 4A). CORT significantly increased *Firmicutes*, *Verrucomicrobiota*, and *Proteobacteria* relative to controls, whereas PHZ recovered these levels and prominently lessened the Firmicutes/Bacteroidetes ratio, in a dose-dependent manner (Figure 4B). At the family level, *Muribaculaceae*, *Bacteroidaceae*, *Prevotellaceae*, *Lachnospiraceae*, *Akkermansiaceae*, and *Lactobacillaceae* were the dominant families (Figure 4C). Compared with the CORT group, PHZ treatment significantly lessened the RAs of *Akkermansiacea*, *Bacteroidaceae,* and *Lachnospiraceae*, whereas it markedly raised the abundance of *Muribaculaceae* (Figure 4D). At the genus level, the top 20 genera by RAs (Figure 5A) encompassed 13 genera whose relative abundance trends were reversed by PHZ treatment (Figure 5B). In detail, *Alloprevotella* and *Lachnospiraceae NK4A136* group significantly increased, whereas *Bacteroides*, *Lachnoclostridium*, *Akkermansia*, *Hungatella*, and *Blautia* decreased. Finally, the PICRUSt2 analysis for forecasting the functional profiles of the gut microbial community exhibited several remarkable disparities between groups at KEGG levels. Specifically, 20 mg/kg was chosen as the low dose for PICRUSt2 experiments, as it represents the minimum effective dose with anti-inflammatory and antidepressant effects, while avoiding high-dose confounding factors. The results of the PICRUSt2 analysis indicate that PHZ may influence metabolic pathways by affecting the microbiota, such as the pentose phosphate pathway, pentose metabolism, glucuronidation, cysteine and methionine metabolism, and fatty acid biosynthesis (Figure 6).

### 2.4. PHZ Changed the Metabolism in CORT Mice

**Figure 7:**
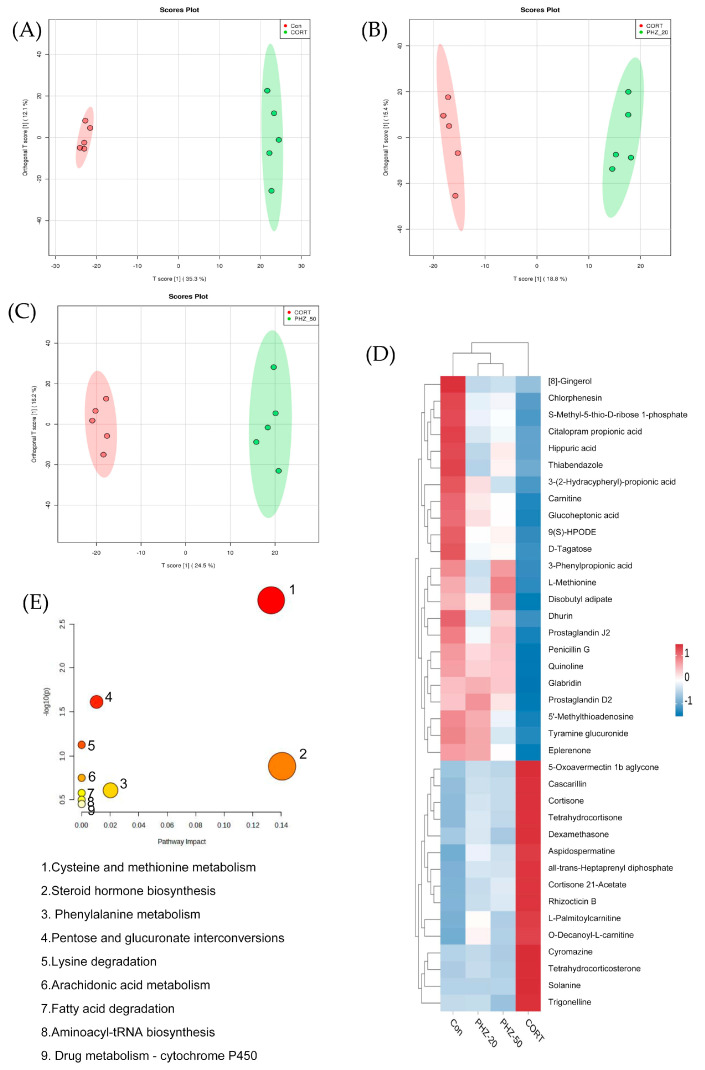
Phlorizin (PHZ) modulates serum metabolomic profiles and associated metabolic pathways in corticosterone (CORT)-induced depressive mice. (**A**–**C**) Orthogonal partial least squares discriminant analysis (OPLS-DA) score plots demonstrating distinct metabolic clustering among groups: (**A**) Control vs. CORT; (**B**) CORT vs. PHZ 20 mg/kg (PHZ-20); (**C**) CORT vs. PHZ 50 mg/kg (PHZ-50). (**D**) Heatmap of 38 significantly altered serum metabolites across groups, clustered using Euclidean distance. (**E**) Bubble plot of metabolic pathway enrichment analysis based on these differential metabolites. Node size indicates the number of metabolites involved in each pathway. *n* = 5.

Given the significant role of metabolites in host–microbiota communication, we analyzed serum metabolites. Orthogonal Projections to Latent Structures Discriminant Analysis (OPLS-DA) revealed clear separation between the experimental groups and CORT-treated mice (Figure 7A–C). The score plots of serum metabolites, shown in Figure S1, confirmed the model’s predictive ability for subsequent analysis. Subsequently, 38 differential metabolites were identified (Table S1). CORT reduced the levels of 23 metabolites, including prostaglandin 2, L-Met, and L-palmitoylcarnitine, while significantly increasing the levels of 15 metabolites, such as N-arachidonoyl glycine, N-palmitoyl leucine, trigonelline, and cortisone acetate. PHZ significantly modulated the abundance of these metabolites (Figure 7D). Further metabolic pathway analysis indicated that PHZ affected nine key metabolic pathways, including cysteine and methionine metabolism, phenylalanine metabolism, steroid hormone biosynthesis, pentose and glucuronate interconversions, arachidonic acid metabolism, fatty acid degradation, aminoacyl-tRNA biosynthesis, and drug metabolism-cytochrome P450 (Figure 7E). Notably, cysteine and methionine metabolism exhibited the highest pathway impact value and was regarded as the most critical pathway.

**Figure 8:**
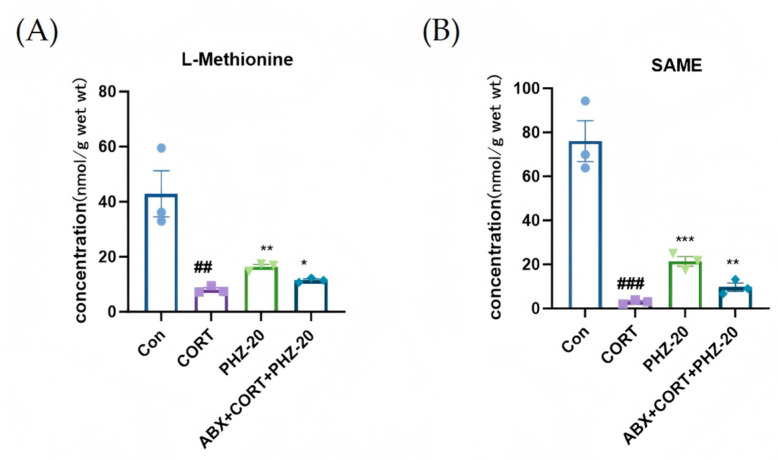
Effect of phlorizin (PHZ) on (**A**) methionine and (**B**) S-adenosylmethionine (SAMe) levels in hippocampal tissues of corticosterone (CORT)-treated mice. Data are expressed as mean ± SEM, *n* = 3. ## *p* < 0.01, ### *p* < 0.001, compared with Control group; * *p* < 0.05, ** *p* < 0.01, *** *p* < 0.001 compared with CORT group.

We further explored whether PHZ can modulate the Met metabolism in brain tissue. To this end, we conducted targeted metabolomics analysis on hippocampal tissue, as the hippocampus is highly susceptible to inflammatory damage and is crucial for emotional regulation. This allowed us to investigate the effects of PHZ on Met metabolism and the potential role of microbial depletion in this process. Figure 8 shows that PHZ-20 prominently raised the levels of SAMe and L-MET in the hippocampus. These two substances are key regulatory factors of Met metabolism and are closely related to neuroinflammation [23]. ABx treatment significantly weakened this effect. These results suggest that PHZ-mediated regulation of metabolites may contribute to its anti-inflammatory effects, and that the gut microbiota plays a pivotal role in this metabolic regulation.

### 2.5. Met Modulates LPS-Induced Inflammation and Promotes M2 Polarization in BV2 Microglial Cells

**Figure 9:**
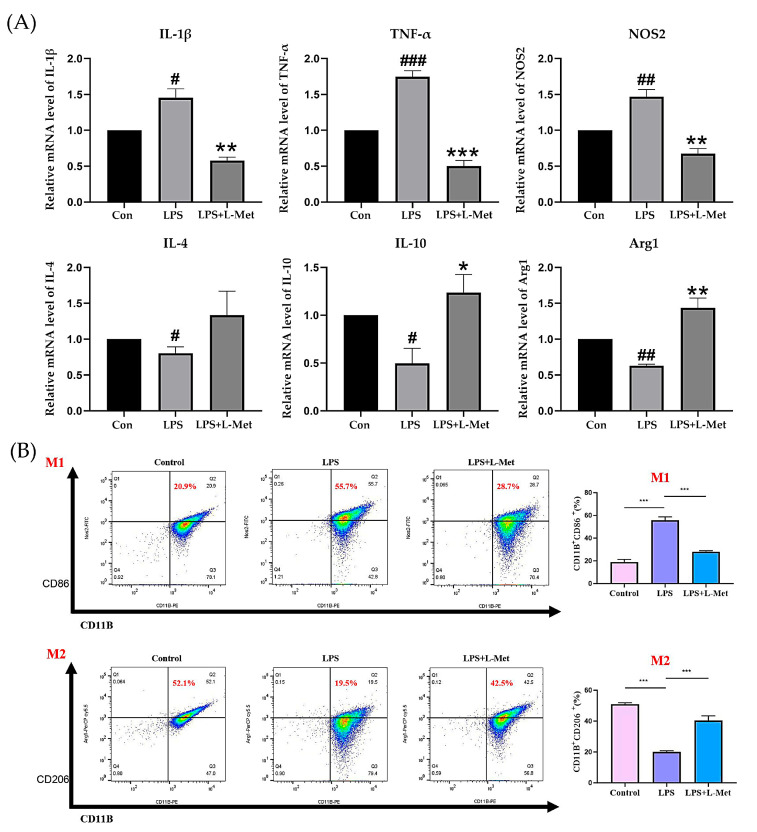
Effect of L-methionine (L-Met) on lipopolysaccharide (LPS)-induced polarization of BV2 microglial cells. (**A**) Gene expression levels of IL-1β, TNF-α, NOS2, IL-4, IL-10, and Arg1 were determined by real-time reverse transcription polymerase chain reaction (RT-PCR). (**B**) CD86 and CD206 expression was assessed by flow cytometry. Data are expressed as mean ± SEM, *n* = 3. # *p* < 0.05, ## *p* < 0.01, ### *p* < 0.001 compared with the control group. * *p* < 0.05, ** *p* < 0.01, *** *p* < 0.001 compared with the LPS-treated group.

To comprehensively explore the anti-inflammatory properties of L-Met, we treated LPS-induced BV2 microglial cells with L-Met and assessed its effect on microglial polarization and production of proinflammatory mediators. BV2 cell viability remained unaffected after treatment with L-Met (Figure S2). LPS stimulation significantly raised the yield of proinflammatory cytokines, like IL-1β, TNF-α, and NOS2, indicating an M1 (pro-inflammatory) polarization of microglia. In contrast, levels of M2 anti-inflammatory cytokines, like IL-4, IL-10, and Arg1, were remarkably reduced following LPS exposure. Importantly, L-Met treatment effectively inhibited the synthesis of LPS-induced M1 pro-inflammatory cytokines (IL-1β, TNF-α, and NOS2) while facilitating the production of M2 anti-inflammatory cytokines (IL-4, IL-10, and Arg1) (Figure 9A). Results suggest that L-Met reduces the inflammatory response and shifts microglial polarization towards the anti-inflammatory M2 phenotype. Flow cytometry deeply confirmed its regulatory effect on microglial phenotype (Figure 9B). These findings provide cellular-level evidence supporting the notion that L-Met, a key metabolite in the Met pathway, regulates microglial polarization and inflammation. This mechanism accords with our previous animal findings that PHZ regulates inflammatory markers and microglial activation, indicating that L-Met is a central mediator of the MGB in its antidepressant action.

### 2.6. Integrative Effect of PHZ on the Microbiome–Metabolism–Brain Axis

**Figure 10:**
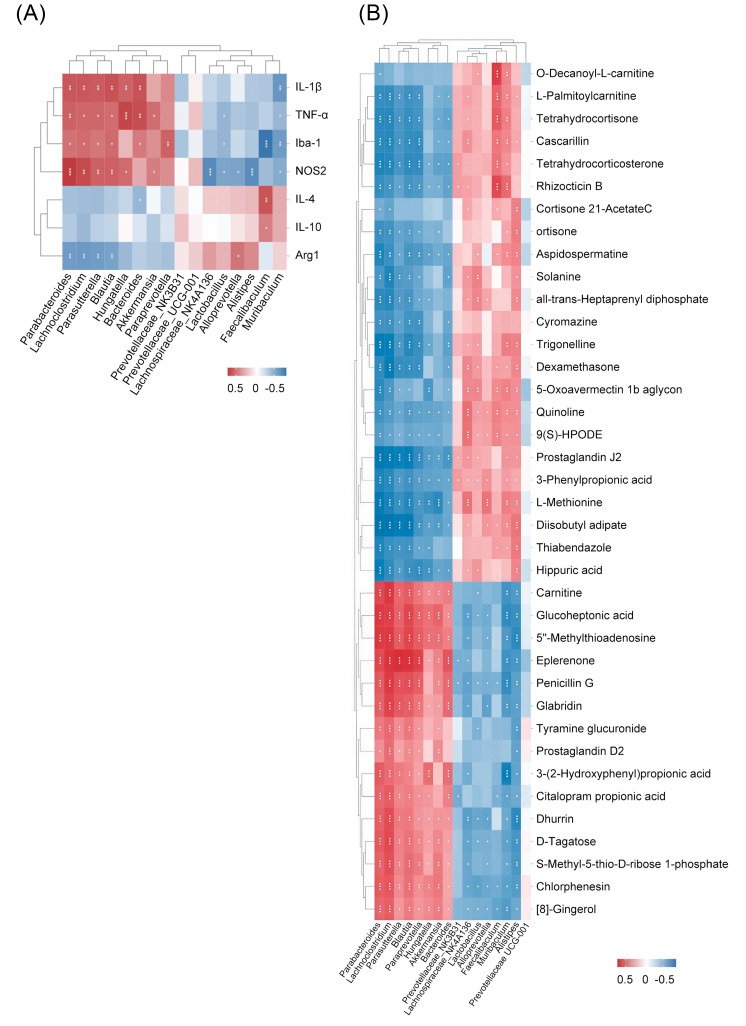
Spearman’s correlation analysis of gut microbiota with cytokines and serum metabolites in corticosterone (CORT)-treated mice with or without phlorizin (PHZ) intervention. (**A**) Heatmap showing Spearman’s correlations between the relative abundances of gut microbiota genera and levels of inflammatory cytokines including IL-1β, TNF-α, IL-4, and IL-10. Red/blue = positive/negative correlations. (**B**) Association map of gut microbiota-serum metabolite relationships. Color intensity reflects correlation strength (red = negative; blue = positive); * *p* < 0.05, ** *p* < 0.01, *** *p* < 0.001.

To systematically construe the correlations among inflammatory factors, serum metabolites, and intestinal microbial community, a correlation matrix was produced via computing the Spearman’s correlation coefficient (Figure 10). Notably, at the genus level, *Parabacteroides*, *Lachnoclostridium*, *Parasutterella*, *Blautia*, *Hungatella*, and *Bacteroides* proactively relate to pro-inflammatory factors, while *Muribaculum* is negatively linked to pro-inflammatory factors. Regarding correlations between serum metabolites and gut microbiota, L-Met was associated with all fungal genera. Overall, these findings support the key role of PHZ in modulating the gut–metabolite axis, thereby contributing to its antidepressant effects.

## 3. Discussion

We explored the antidepressant effects of PHZ and its underlying mechanisms in a CORT-induced mouse model of depression. Following five weeks of PHZ gavage in CORT mice, our findings demonstrated that PHZ increased body weight, effectively ameliorated depressive-like behaviors, modulated the microglia M1/M2 polarization balance, and ameliorated hippocampal neuroinflammation. This highlights that PHZ, as a safe and effective Intervention, holds potential for preventing depression.

Microglia are a pivotal player in the etiology of depression [10]. Brain microglia become activated under stress and polarize into either M1 or M2 phenotypes. The M1 pro-inflammatory phenotype is typically linked to inflammatory responses and neurotoxicity, leading to neuronal damage and neurotransmitter imbalances that exacerbate the severity and relapse of depression [24]. Nevertheless, the M2 anti-inflammatory phenotype exhibits anti-inflammatory and reparative effects by secreting anti-inflammatory cytokines and neurotrophic factors, thereby promoting neuroprotection and recovery of brain function [24]. Accordingly, modulating microglial polarization phenotypes serves as an effective strategy for alleviating neuroinflammation-related depressive behaviors [10]. In this study, PHZ suppressed M1 polarization and pro-inflammatory cytokine expression, while promoting M2 polarization and increasing IL-4 and IL-10 levels. These results demonstrate that PHZ shifts microglial polarization toward an anti-inflammatory profile, consistent with previous findings in HFFD mice [16]. Research has shown that there are significant changes in gut microbiota diversity in CORT mice [25]. Importantly, gut microbiota dysbiosis is closely related to the M1 polarization of microglia [26]. Results indicate that PHZ predominantly raised the abundance of anti-inflammatory bacteria and decreased that of pro-inflammatory bacteria. The abundance of *Lachnoclostridium*, *Blautia*, and *Bacteroides* increased can trigger gut inflammation and disrupt the intestinal barrier [27]. Conversely, PHZ increased the RA of *Alistipes*, *Alloprevotella*, and *Lachnospiraceae NK4A136* group. Notably, *Alistipes* abundance has been negatively correlated with depressive behaviors and mucosal barrier damage, while the butyrate-producing *Lachnospiraceae NK4A136* group has been linked to improved gut integrity, depression recovery, and brain function [27,28,29,30]. Together, these findings suggest that alterations in the abundance of these key bacterial taxa significantly affect the gut mucosal barrier and nervous system, and that PHZ’s pronounced effects on these bacteria highlight its anti-inflammatory role. Nevertheless, as only 16S rRNA sequencing was applied in this study, our findings provide community-level insights but cannot resolve species-level or functional information. Future metagenomic analyses will be required to identify the specific microbial taxa and pathways involved.

Small-molecule metabolites serve as the link between gut microbiota and brains [31]. Serum metabolomics analysis revealed that PHZ significantly modulated Met metabolism. Met becomes a critical sulfur-containing amino acid extensively involved in physiological activities, like lipid metabolism, innate immune reactions, and oxidative stress [32]. The disruption of methionine fine metabolism is associated with neurological and psychiatric disorders. In this study, the serum levels of L-Met and its metabolites were prominently decreased in the CORT group, whereas PHZ treatment elevated their levels. Quantitative hippocampal analysis further demonstrated that PHZ treatment significantly increased hippocampal levels of L-Met and SAMe, suggesting that Met metabolism is a pivotal player in the antidepressant effects of PHZ. Elevated blood Met levels (a direct precursor to SAMe) can in turn rapidly increase Met and SAMe levels in the brain, thus exerting antidepressant effects [33]. As a key methyl donor, Met can also regulate the polarization state of microglia [34], effectively alleviating neuroinflammatory responses and improving cognitive function [23]. However, some other studies have also indicated that long-term or high-dose L-Met can exacerbate neuroinflammation and impair neurogenesis [35]. In our research, PHZ increased hippocampal methionine content and elevated SAME levels in mice with mildly elevated CORT, thereby alleviating neuroinflammation. Moreover, within the concentration range tested in vitro (up to 10 mM), L-Met had no significant impact upon the activity of BV2 cells, further supporting the safety of this moderate level of L-Met. Notably, gut microbiota-mediated Met metabolism has been regarded as an underlying target for treating Met metabolism-related disorders [36]. For instance, *Akkermansia* regulates methionine metabolism and increases the gut microflora among Parkinson patients [37]. A high-Met diet changes the abundance of specific gut microbe strains (e.g., increasing *Lachnospiraceae* and *Alistipes*, decreasing *Faecalibaculum*), leading to elevated circulating homocysteine levels in mice [38]. Furthermore, Met supplementation can alleviate gut inflammation by modulating the abundance of strains, such as *Parabacteroides*, subsequently influencing central nervous system function [39].

Our study discovered that elevated serum Met levels negatively related to the abundance of *Parabacteroides* following PHZ administration in CORT, and actively related to the abundance of *Lachnospiraceae NK4A136* group and *Alistipes*, consistent with these previous data. Furthermore, ABX significantly reduced the increase in methionine levels after PHZ administration. This further suggests that PHZ may regulate Met metabolism by modulating the gut microorganisms among CORT mice. Beyond the gut microbiota–methionine–neuroinflammation pathway, PHZ may also exert antidepressant effects through additional mechanisms. As a well-known glucose transporter inhibitor, PHZ can modulate systemic and brain glucose metabolism, a process closely linked to mood regulation. Moreover, PHZ has antioxidant properties that may protect neurons from oxidative-stress-induced damage. Similar antidepressant effects have been reported for other plant-derived polyphenols and flavonoids, such as resveratrol and quercetin, which act on the MGB and neuroinflammation. However, PHZ may provide unique benefits due to its dual action on gut microbiota and glucose transport. Future studies should systematically evaluate these alternative pathways to clarify their relative contributions to PHZ’s antidepressant efficacy. However, this study has several limitations. First, the precise mechanisms by which gut microbiota regulate methionine metabolism remain to be clarified. Second, while the CORT-induced depression model is a classical paradigm, it cannot fully recapitulate the complexity of human depression. Moreover, although our 5-week study indicated that PHZ was well tolerated at the tested doses, its long-term efficacy and potential adverse effects under chronic administration require further evaluation. Finally, it is worth noting that given its action on the gut–brain axis, PHZ could theoretically be used in combination with selective serotonin reuptake inhibitors (SSRIs) or serotonin–norepinephrine reuptake inhibitors (SNRIs); thus, future clinical studies should investigate both monotherapy and combination strategies to determine the optimal therapeutic regimen.

## 4. Materials and Methods

### 4.1. Materials

Shanghai Yuanye Biotechnology Co. (Shanghai, China) supplied the PHZ. Sertraline was purchased from Guang’anmen Hospital, China Academy of Chinese Medical Sciences (Beijing, China) (Zhejiang Jingxin Pharmaceutical Co., Ltd., Shaoxing, China, National Medicine Standard H20051076; specification: 50 mg). SAME (S5109) and L-Met (S5633) were purchased from Selleck (Selleck Chemicals, Houston, TX, USA). CORT (S30265) was purchased from Shanghai Yuanye Bio-Technology Co., Ltd (Shanghai, China). Neomycin sulfate (B540734), Ampicillin (A430258), Vancomycin hydrochloride (A430243), and Metronidazole (A429689) were purchased from Sangong Biotechnology (Shanghai, China). DAPI (S2110) was purchased from Solarbio (Beijing, China). Iba-1 (ab178846), NOS2 (ab178945), antibodies against alpha tubulin (ab176560), and FITC-labeled secondary antibodies (ab6717) were purchased from Abcam (Cambridge, UK). Antibodies against caspase 1 (sc-56036) were obtained from Santa Cruz Biotechnology, Inc. (Dallas, TX, USA); antibodies against Arg1 (66129-1-l) were obtained from Proteintech Group, Inc. (Wuhan, China); CD86 (12-0862-82), CD206 (17-2061-82), CD11b (17-0112-83), and ELISA kits of IL-10 (BMS607-3), IL-4 (88-7044-88), IL-1β (BMS6002), and TNF-α (BMS607-3) were obtained from Thermo Fisher Scientific (Waltham, MA, USA); Antibodies against GAPDH (G0100) were obtained from Lablead Biotech Co., Ltd. (Beijing, China); CCK-8 (C0037; Beyotime, Shanghai, China), EZ-press RNA Purification Kit (B0004D; EZBioscience, Roseville, CA, USA, USA), Color Reverse Transcription Kit (A0010CG; EZBioscience, Roseville, CA, USA), Color SYBR Green qPCR Master Mix (A0012-R2; EZBioscience, Roseville, CA, USA). BV2 mouse microglial line, fetal bovine serum (FBS), and 1% penicillin-streptomycin were supplied by ScienCell (Roseville, CA, USA). Cell Counting Kit-8 assay (CCK-8) was provided by Dojindo (Mashiki, Japan).

### 4.2. Effect of PHZ on Weight and Behavior in CORT-Induced Depression Mice

#### 4.2.1. Depression Modeling and Drug Administration

Provided by Charles River Laboratories (Beijing, China), seven-week-old male C57BL/6 mice were raised within one ventilated animal compartment at 23  ± 1 °C and 55–65% relative humidity during a 12-h light/dark cycle. Except for the sucrose preference test (SPT), animals were provided chows and water for free. The experimental protocols abided the guidelines of the Committee for Animal Care and Use of Laboratory Animals at the Beijing University of Traditional Chinese Medicine (permission number BUCM-4-2021083003-3086).

**Figure 11:**
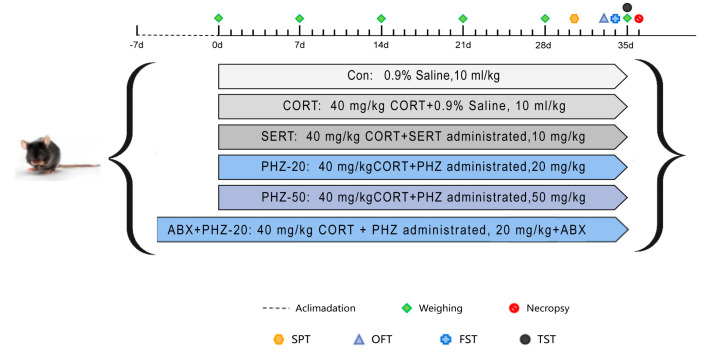
Experimental design.

After a seven-day acclimatization period, mice were randomly allocated to five groups (*n* = 10): control, CORT, sertraline, PHZ 20 mg/kg (PHZ-20), and PHZ 50 mg/kg (PHZ-50). Grouping was performed using an online randomization tool (http://www.randomizer.org/, accessed on 10 October 2024), and the researchers were blinded to the group allocation during the experiments. The doses of PHZ were selected based on existing literature. The literature indicates that 20 mg/kg and 50 mg/kg can demonstrate their efficacy in modulating gut microbiota and alleviating neuroinflammation [15,16]. CORT was digested in physiological saline (Laboratorios ERN, Barcelona, Spain) comprising 0.1% Tween-80 and 0.1% dimethyl sulfoxide (Laboratorios ERN, Barcelona, Spain) to make a 2 g/L solution uniformly mixed using ultrasonic vibration (Scientz, Wuxi, China). Throughout the experiment, all mice, except those in controls, were injected with CORT at a dose of 40 mg/kg each day for 5 weeks. The controls received physiological saline injections at the identical dose and timing. The drug was administered intragastrically to the treatment groups 30 min before CORT injection. The PHZ powder was completely dissolved in 0.1% dimethyl sulfoxide. The mixture was diluted with double-distilled water to a concentration of 5 mg/mL. Drugs were administered by gavage to the PHZ-20 and PHZ-50 groups at doses of 20 and 50 mg/kg, respectively. Medications were intragastrically administered from the first week of treatment before the CORT injections. The doses used for each group are shown in Figure 11. Antibiotic treatment experiments were used to verify that the gut microorganisms mediated the PHZ’s neuroprotection of key metabolites in a mouse model of CORT.

In water, the final concentrations of antibiotics are listed as follows: ampicillin (1 g/L), vancomycin (0.5 g/L), neomycin (1 g/L), and metronidazole (1 g/L). The solution was replaced every 3 days to sustain its effectiveness. On day 8, the mice were modeled and gavaged with the same PHZ-20 and received water containing antibiotic solution throughout.

#### 4.2.2. Behavioral Testing

Behavioral tests, including body weight measurement, FST, SPT, OFT, and TST were executed at fourth week post-modeling. Animals acclimated for 1 h prior to testing to reduce stress-induced variations. All behavioral tests were implemented at the identical time of day, in the afternoon.

Body weight: Growth ratios were calculated using Equation (1), from weekly measurements obtained using electronic scale weights:

(1) $$Growth\;ratio\left(\%\right)=\frac{\left(weight\;after\;modelling-weight\;before\;modelling\right)}{weight\;before\;modelling}\times 100$$

SPT: The primary objective was to determine anhedonia levels in the animals. The mice were provided with two indistinguishable vials comprising a 1% sucrose solution for 24 h. Subsequently, this solution was replaced by water. Following 12 h, the locations of these two vials were exchanged to remove any preference for a certain position. The mice were introduced to sucrose solution and water for 4 h and were subsequently weighed. The SP values were computed using Equation (2) [40]:

(2) $$SP\left(\%\right)=\frac{sucrose\;intake(vol)}{\left[sucrose\;intake\left(vol\right)+water\;intake(vol)\right]}\times 100$$

OFT: Three minutes after being positioned in the middle of the open ground, mice were allowed to roam freely. Following adaptation, the distance traveled by each mouse within the subsequent 3 min was documented. Prior to evaluating the mice, the handler sterilized the OFT apparatus with a 75% ethanol solution [41].

FST: A hyaline cylinder (18 × 30 cm) was filled with 20 cm of water at 25 ± 1 °C. Mice were observed for 6 min. During the final 4 min, immobility time was recorded. Water was replaced after each test [42].

TST: Each mouse was hung by its tail tip, roughly 1 cm from the end, for 6 min using adhesive tape. The cumulative stationary time after the 2 min regulation period was recorded. When the mice ceased their rapid movements and merely moved to breathe, they were regarded as immobile. The observers did not know about the group of mice [43].

### 4.3. Enzyme-Linked Immunosorbent Assay (ELISA)

Mouse TNF-α, IL-1β, IL-4, IL-6, and IL-10 levels in the hippocampal supernatant were quantified through ELISA kits complying with the manufacturer’s protocols.

### 4.4. Immunofluorescence

During general anesthesia with 1% pentobarbital sodium (50 mg/kg; Sigma-Aldrich, St. Louis, MO, USA), mouse chest was opened, the heart exposed, and normal saline solution was injected into the heart tip. Following the removal of fluids and the absence of any blood color, whole brain tissue was extracted from the decapitated head and then immersed in 4% paraformaldehyde (Solarbio, Beijing, China) for extra 24 h. The brain specimens were dried with various alcohol concentrations, treated with xylene, and then immobilized in paraffin. Subsequently, the brain samples were cut into consecutive coronal slides, each measuring 5 μm in thickness. Tissue slices were subjected to antigen retrieval by placing them in citric acid antigen repair solution (pH 6.0; Solarbio, Beijing, China). Subsequently, they were treated with the primary antibody Iba-1 (1:2000) in a humid compartment throughout the night at 4 °C. The subsequent day, the slices were cleaned using PBS, subsequently processed with drops of an FITC-conjugated secondary antibody (1:3000), before cultivation for 50 min and away from light. After restaining through DAPI (1:100) for 10 min and washing through PBS, an antifluorescence quenching sealing agent (Solarbio, Beijing, China) was utilized. Finally, slides were observed under a fluorescence microscope (Olympus, Tokyo, Japan) [44,45].

### 4.5. Western Blot

Total proteins from the hippocampus were obtained by a RIPA lysis buffer that contained a 1× protease inhibitor cocktail. To determine the concentration of proteins, a BCA assay test kit (Thermo Fisher Scientific, Waltham, MA, USA) was employed as per the manufacturer’s guidelines. To sum up, the proteins underwent separation via a 10% SDS-PAGE gel and were subsequently delivered onto a 0.45 µm PVDF membrane (Millipore, Billerica, MA, USA). Such membranes were then cultivated using 5% non-fat dried milk for 1 h at 23 °C, before an all-night incubation at 4 °C using primary antibodies targeting NOS2 (1:500), Arg1 (1:5000), and alpha tubulin (1:5000). After performing three washes with TBST, the membranes were nurtured using the appropriate goat antirabbit IgG-HRP secondary antibody (1:10,000) for 1 h at 25 °C. Another wash with TBST was performed, after which the ECL reagent was used for observing immunoreactivity. Afterward, such membranes were scanned with ChemiDoc Imagers (BioRad, Hercules, CA, USA). Concurrently, band intensity was assessed via ImageJ software (version 1.52a, National Institutes of Health, Bethesda, MD, USA) [46].

### 4.6. 16S rRNA Gene Sequencing

To assess variations of gut microflora, stool samples from five mice per group underwent 16S rRNA sequencing. After DNA extraction, the DNA was detected through 1% agarose gel electrophoresis. Pure DNA underwent polymerase chain reaction amplification as per the manufacturer’s guidelines. Sequences that had been optimized through sequence splicing and filtration were obtained, and chimeric reads were removed. Libraries were sequenced through the MiSeq PE300 platform (Illumina, San Diego, CA, USA). 

Bacterial diversity was appraised by means of alpha diversity indicators, like Chao1, PD, Shannon, and observed species, as well as principal coordinate analysis (PCoA) on the grounds of Bray-Curtis distances. Enriched bacteria were recognized with linear discriminant analysis (LDA) through LEfSe. Additionally, differential bacterial species were gauged using *p*  ≤  0.05. The prediction spectrum for gene function within the bacterial domain was constructed using PICRUSt2 (https://github.com/picrust/picrust2, accessed on 5 January 2025) [47].

### 4.7. Metabolomics Analysis

#### 4.7.1. UPLC-Q-TOF/MS Detection Analysis

An Eppendorf (EP) tube (Eppendorf, Hamburg, Germany) was utilized to collect 100 μL serum sample, which was then treated with an extract solution (acetonitrile:methanol = 1:1, *v*/*v*; Thermo Fisher Scientific, Waltham, MA, USA), containing isotopically labeled internal standard mixture). The materials were vigorously mixed for 15 s using a vortex (Wiggens, Beijing, China), followed by centrifugation (12,000 rpm for 10 min at 4 °C). The supernatant was transmitted into a 200 μL container for analysis via ultra-high-performance liquid chromatography (Waters Corp., Milford, MA, USA) incorporated into quadruple time-of-flight mass spectrometry (UPLC-Q-TOF/MS) (Waters Corp., Milford, MA, USA).

#### 4.7.2. Differential Metabolites Identification and Data Analysis

Raw data were processed using Analyst software (version 1.4.2; AB Sciex Inc., Concord, ON, Canada). for peak alignment, integration, and normalization. Features consistently detected across samples were retained for further analysis. Multivariate analyses, including PCA, PLS-DA, and OPLS-DA, were performed using MetaboAnalyst 6.0 and R (v4.3.2) to evaluate metabolic differences among groups. Differential lipid metabolites were identified based on VIP > 1.0 and *p* < 0.05 (two-tailed *t*-test). Metabolites were annotated using HMDB (http://www.hmdb.ca, accessed on 28 January 2025) and KEGG http://www.genome.jp/kegg/, accessed on 28 January 2025) based on mass accuracy and MS/MS spectra. Pathway enrichment and network analysis were conducted using MetaboAnalyst (http://www.metaboanalyst.ca/MetaboAnalyst/, accessed on 28 January 2025), referencing KEGG databases. Heatmaps and clustering were generated to visualize lipidomic alterations across groups.

### 4.8. Methionine-Targeted Metabolomics Analysis

Hippocampal tissues were homogenized in pre-cooled methanol (4:1, tissue to methanol, *v/w*) with centrifugation at 13,000 rpm for 15 min at 4 °C. The supernatant was filtrated via a 0.22 μm membrane and stored at −80 °C until analysis. Quantification of SAMe (399.4 g/mol, C15H22N6O5S), and L-Met (149.21 g/mol, C5H11NO2S) was performed using UPLC-MS/MS in multiple reaction monitoring mode. For each metabolite, calibration curves were formulated using standard solutions to ensure specificity and sensitivity. The mobile phase comprised acetonitrile with 0.1% formic acid and water, and the metabolites were separated on a reverse-phase column (Waters ACQUITY UPLC BEH C18, 2.1 × 100 mm, 1.7 μm; Waters Corp., Milford, MA, USA) using a gradient elution program. Metabolite concentrations were normalized to the tissue weight for comparative analysis.

### 4.9. Cell Viability Assay

Cell activity was appraised through the CCK-8 assay, performed on a 96-well plate (Corning Inc., Corning, NY, USA) [48]. Briefly, BV2 cells were plated and handled using discrepant concentrations of L-Met, both with and without LPS treatment, for a 24 h period. Afterwards, 10 μL CCK-8 reagent was supplemented to each well. The absorbance at 450 nm was gauged through a microplate reader (Thermo Fisher Scientific, Waltham, MA, USA) after a 2 h incubation at 37 °C.

### 4.10. Cell Culture and Treatment

The immortalized BV2 microglial cell line derived from mice was nurtured within MEM medium comprising 10% FBS and 1% penicillin/streptomycin. Such cells were preserved within a humid brooder (Thermo Fisher Scientific, Waltham, MA, USA) under 95% air and 5% CO_2_ at 37 °C. The medium was displaced every other day. After reaching 80% confluence, the cells were isolated through 0.25% trypsin and passaged for continued propagation. For the experimental procedures, cells were handled with 10 mM Met for 16 h, before exposed to LPS (500 ng/mL) for 4 h.

### 4.11. Quantitative Real-Time PCR

According to the manufacturer’s specifications, total RNA was obtained from each specimen through the EZ-press RNA Purification Kit (EZBioscience, Roseville, CA, USA). Complementary DNA synthesis was performed through the Color Reverse Transcription Kit, with the final reaction volume adjusted to 20 μL. Quantitative real-time PCR (qPCR) was executed thrice through the 2× Color SYBR Green qPCR Master Mix. As for all genes, the mRNA levels were standardized to the β-actin mRNA levels as a reference. Used primers in are listed in Table 1.

### 4.12. Flow Cytometry

Cell surface markers CD11b, CD86, and CD206 were detected via flow cytometry. Briefly, after BV2 cells were treated as described in Section 4.10, cells were harvested through EDTA-free trypsin, underwent centrifugation at 1000 rpm for 5 min, and were rinsed twice using cold PBS to collect cell deposits. Subsequently, such cells were suspended in 100 μL PBS again and stained with anti-CD11b (1:80), anti-CD206 (1:160), and anti-CD86 (1:300) antibodies at 4 °C for 20 min. All specimens were construed via imaging flow cytometry (ImageStream^®^X Mark II; Luminex Corp., Austin, TX, USA). In addition, data analytics was performed using IDEAS software (version 6.2; Luminex Corp., Austin, TX, USA).

### 4.13. Statistical Analysis

All data are denoted as mean ± standard error. The data was visualized through GraphPad Prism (v 6.01) (GraphPad, San Diego, CA, USA). One-way analysis of variance was used for appraising data significance, before a post-hoc Student–Newman–Keuls test and an unpaired two-tailed Student’s *t*-test. In this study, *p* < 0.05 held statistical significance. The significance levels were denoted as * *p* < 0.05, ** *p* < 0.01, and *** *p* < 0.001. The differences in effect size ratios of the linear discriminant analysis on core flora between the groups were rated through the non-parametric Kruskal–Wallis rank sum test. Spearman’s correlation was adopted to evaluate the interrelationships among parameters.

## 5. Conclusions

**Figure 12:**
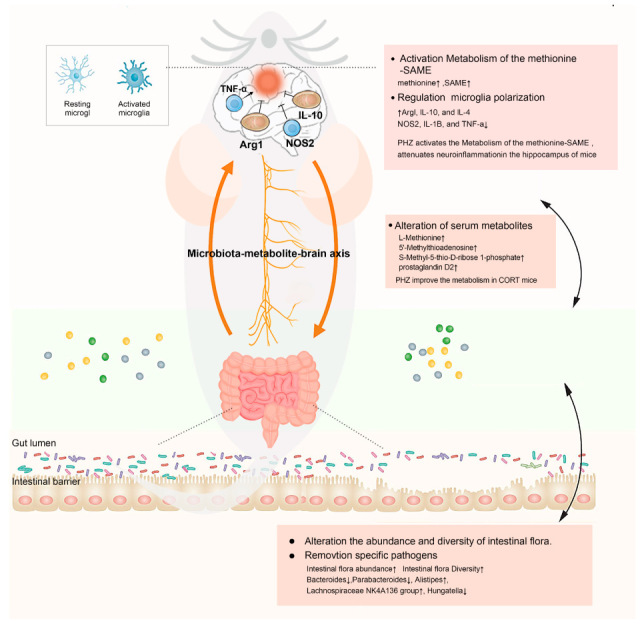
A schematic diagram of the potential mechanism of the antidepressant effect of phlorizin (PHZ). In addition to microglia-mediated neuroinflammation, the gut–brain axis is an important target for the beneficial effects of PHZ.

Our findings demonstrate that PHZ ameliorates depressive states and exerts anti-inflammatory effects in CORT mice. Such an effect is likely connected to the regulation of gut microbial community abundance by PHZ, which alters the serum levels of key metabolites, including Met. This process subsequently elevates central Met levels. Met acts as a key mediator of “periphery-to-center” pharmacological effects of PHZ, modulates the M1/M2 polarization balance of microglia, suppresses neuroinflammation, and consequently improves CORT-induced depressive-like behaviors in mice (Figure 12).

## Figures and Tables

**Table 1:** Primer sequences for each gene analyzed by qPCR.

Gene	Forward (5′–3′)	Reverse (5′–3′)
IL-1β	TTTGAAGTTGACGGACCCCAA	CACAGCTTCTCCACAGCCACA
IL-4	ATCGGCATTTTGAACGAGGTCACA	CGAAGCACCTTGGAAGCCCTA
IL-10	TTACCTGGTAGAAGTGATGCCC	GACACCTTGGTCTTGGAGCTTA
Arg-1	AGGAAAGCTGGTCTGCTGGAA	AGATGCTTCCAACTGCCAGAC
NOS2	GGGCTGTCACGGAGATCAATG	GCCCGGTACTCATTCTGCATG
TNF-α	ACCACGCTCTTCTGTCTACT	AGGAGGTTGACTTTCTCCTG
β-actin	CTGAGAGGGAAATCGTGCGT	CCACAGGATTCCATACCCAAGA

## Data Availability

The original contributions presented in the study are included in the article/Supplementary Materials; further inquiries can be directed to the corresponding authors.

## Supplementary Materials

The following supporting information can be downloaded at: https://www.mdpi.com/article/10.3390/ph18091395/s1, Figure S1: Validation plots for each combination of OPLS-DA models; Figure S2: Effects of L-Met on BV2 microglial cell viability. Table S1: List of 38 differential serum metabolites identified between experimental groups.

## Abbreviations

The following abbreviations are used in this manuscript:

ABx	antibiotic mixture
CCK-8	Cell Counting Kit-8 assay
CORT	corticosterone
ELISA	enzyme-linked immunosorbent assay
EP	Eppendorf
F/B	Firmicutes/Bacteroidetes
FST	forced swimming test
HFFD	high-fat, high-fructose diet
Ig	immunoglobulin
IL	interleukin
L-Met	L-methionine
LPS	lipopolysaccharide
OPLS-DA	Orthogonal Projections to Latent Structures Discriminant Analysis
PHZ	Phlorizin
SAMe	S-adenosylmethionine
SPT	sucrose preference test
SSRI	selective serotonin reuptake inhibitors
TNF	tumor necrosis factor
TST	tail suspension test
UPLC-Q-TOF/MS	ultra-high-performance liquid chromatography integrated with quadruple time-of-flight mass spectrometry
VIP	Variable Importance in Projection
MGB	microbiota–gut–brain
RAs	relative abundances
