# Supplementary figures and images for: Phlorizin Alleviates Depression-like Behaviors via Gut Microbiota Reprogramming-Induced Methionine to Inhibit Neuroinflammation in Mice Hippocampus
Source: Pharmaceuticals (Basel). 2025 Sep 17;18(9):1395. doi: 10.3390/ph18091395 (PMC12472783; doi:10.3390/ph18091395)

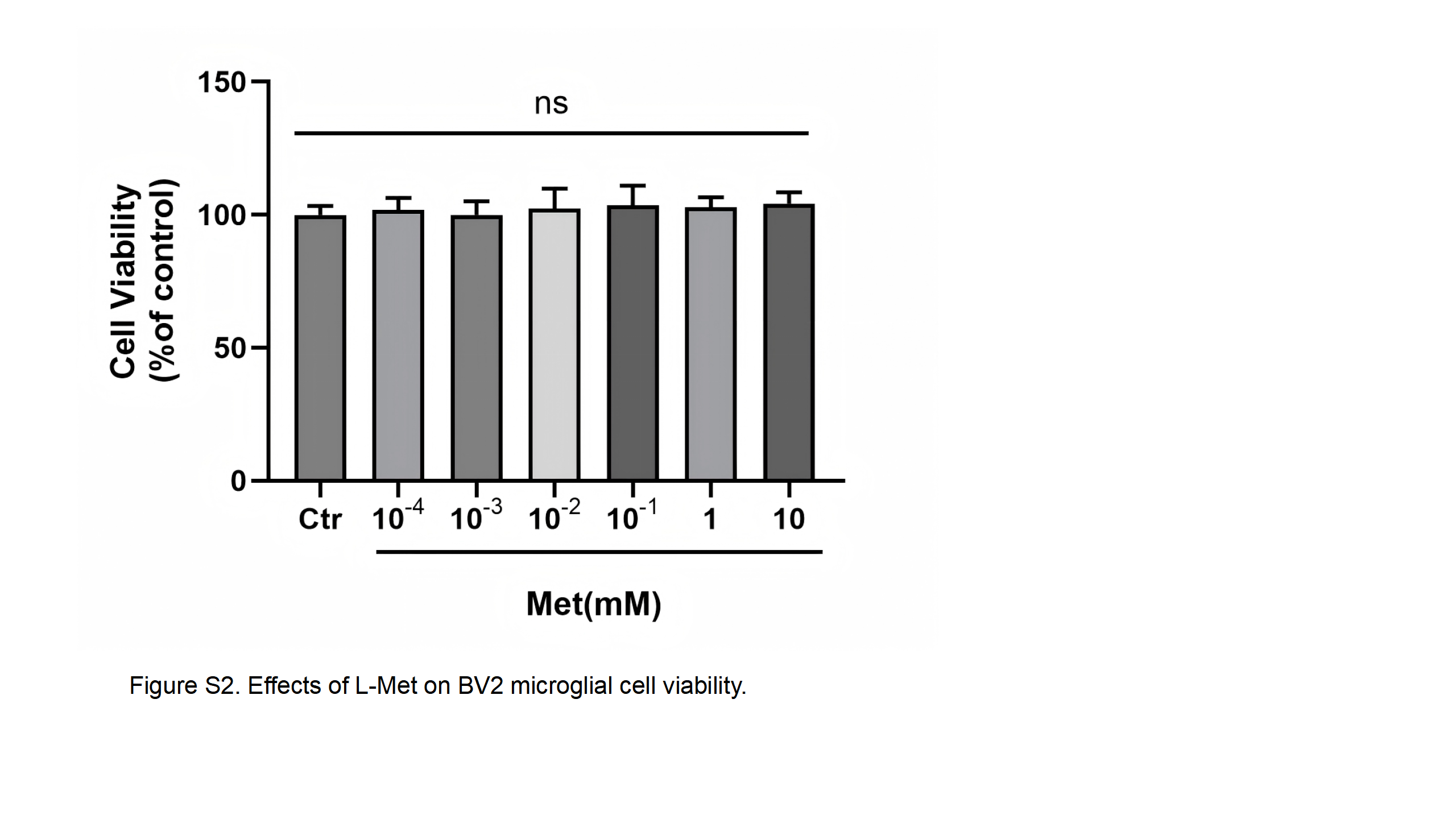

Supplement: Supplementary file 1 [file pharmaceuticals-18-01395-s001.zip › Supplementary File(s)/Figure S2.tif]
